# Inference for Inverse Power Lomax Distribution with Progressive First-Failure Censoring

**DOI:** 10.3390/e23091099

**Published:** 2021-08-24

**Authors:** Xiaolin Shi, Yimin Shi

**Affiliations:** 1 School of Electronics Engineering, Xi’an University of Posts and Telecommunications, Xi’an 710121, China; shixiaolin@xupt.edu.cn; 2 School of Mathematics and Statistics, Northwestern Polytechnical University, Xi’an 710072, China

**Keywords:** inverse power Lomax distribution, progressive first-failure censoring, maximum likelihood estimation, confidence interval, bayesian estimation, Tierney–Kadane’s approximation, credible interval, the importance sampling procedure

## Abstract

This paper investigates the statistical inference of inverse power Lomax distribution parameters under progressive first-failure censored samples. The maximum likelihood estimates (MLEs) and the asymptotic confidence intervals are derived based on the iterative procedure and asymptotic normality theory of MLEs, respectively. Bayesian estimates of the parameters under squared error loss and generalized entropy loss function are obtained using independent gamma priors. For Bayesian computation, Tierney–Kadane’s approximation method is used. In addition, the highest posterior credible intervals of the parameters are constructed based on the importance sampling procedure. A Monte Carlo simulation study is carried out to compare the behavior of various estimates developed in this paper. Finally, a real data set is analyzed for illustration purposes.

## 1. Introduction

In the life test of a product, due to the restrictions of test time, cost and other conditions, the complete life test is not generally performed. In these cases, experimenters often use censoring schemes to obtain censored lifetime data. There are many types of censoring schemes, and the most popular censoring schemes are Type-I and Type-II censoring. In Type-I censoring, the test ends at a pre-fixed time, while in Type-II censoring, the test ends when the m-th failure occurs (m is fixed in advance). For the above two censoring schemes, the common disadvantage is that no unit in the test can be removed before the test is terminated. Thus, progressive censoring (PC) was proposed, which has better efficiency in lifetime experiments. Under this censoring scheme, one can remove the test units at various stages of the experiment. For more details, refer to Balakrishnan and Aggarwala [1]. An excellent review of progressive censoring schemes can be found in Ref. [2]. Besides the PC, there is another censoring scheme, namely the first failure censoring scheme. Under this censoring scheme, experimenters group the test units into several sets and then perform all the test units simultaneously until the first failure in each set. The first-failure censoring scheme was studied by Johnson [3], Balasooriya et al. [4], Wu et al. [5] and Wu and Yu [6]. However, this censoring scheme does not allow the removal of units from the test at points other than the final termination point. Wu and Kus [7] combined the advantages of the first failure censoring and progressive censoring to propose mixed censoring, that is, a progressive first-failure censoring (PFFC) scheme. They obtained maximum likelihood estimates (MLEs), interval estimation and expected time on test for the parameters of the Weibull distribution based on the PFFC sample.

The PFFC scheme can be described as follows: suppose that n independent groups with *k* items within each group are put on a life test at time zero, and the progressive censoring Scheme $\underset{˜}{R}=(R_{1},R_{2},\ldots ,R_{m})$ is fixed in advance. At the first failure time $X_{1:m:n:k}$, $R_{1}$ groups and the group in which the first failure is observed are randomly removed from the test. Similarly, at the second failure time $X_{2:m:n:k}$, $R_{2}$ groups and the group in which the second failure is observed are randomly removed from the remaining $\left(n-R_{1}-1\right)$ groups. This procedure continues until the *m*th failure time $X_{m:m:n:k}$ is observed in the remaining groups, and then all the remaining $R_{m}$ groups are removed. It is clear that $n=m+R_{1}+R_{2}+\ldots +R_{m}$. The observed failure times, $X_{1:m:n:k}<X_{2:m:n:k}$ $<\ldots <X_{m:m:n:k}$, are called the PFFC sample with the progressive censoring scheme $\underset{˜}{R}=(R_{1},R_{2},\ldots ,R_{m})$. Here, (m,n,k) must be pre-specified.

The main advantage of the PFFC scheme is that it reduces time where more items are used, but only *m* out of n×k items are observed. It is observed that if $R_{1}=R_{2}=\ldots =R_{m}=0$, the PFFC reduces to first failure censoring; If k=1, the scheme becomes progressively Type II censoring; when *k* = 1, $R_{1}=R_{2}=\ldots =R_{m-1}=0$ and $R_{m}=n-m$, this scheme reduces to Type II censoring scheme. Furthermore, the progressively first-failure censored sample $X_{1:m:n:k}<X_{2:m:n:k}<\ldots <X_{m:m:n:k}$ can be viewed as a progressively Type-II censored sample from a population with the distribution function $1-{\left(1-F(x)\right)}^{k}$, which enables us to extend all the results on progressive type-II censored order statistics to progressive first-failure (PFF) censored order statistics.

Because of the flexibility of the PFFC scheme, many scholars have discussed and applied it in reliability studies. Ref. [8] studied statistical inferences of the unknown parameters, the reliability and failure functions of the inverted exponentiated Half-Logistic distribution using PFFC samples. Ref. [9] investigated a competing risks data model under PFFC from a Gompertz distribution using Bayesian and non-Bayesian methods. Ref. [10] considered the estimates of the unknown parameters and reliability characteristics of generalized inverted exponential distribution using PFFC samples. Ref. [11] established different reliability sampling plans using two criteria from a Lognormal distribution based on the PFFC. Some recent studies on the PFFC scheme can be found in Refs. [12,13,14,15,16].

The inverse distributions have a wide range of applications in issues related to econometrics, biological sciences, survey sampling, engineering sciences, medical research and life testing problems. In recent years, some scholars have studied the statistical inference of inverse distribution. For example, Dube et al. [10] studied the MLEs and Bayesian estimators of the unknown parameters and reliability characteristics of generalized inverted exponential distribution using progressively first-failure censored samples. Panahi and Moradi [17] discussed the estimation of the inverted exponentiated Rayleigh distribution based on an adaptive Type II progressive hybrid censored sample. Bantan et al. [18] studied the estimation of the Rényi and q-entropies for inverse Lomax distribution under multiple censored data. An efficient estimation strategy was proposed by using the maximum likelihood and plugging methods. But they did not investigate the statistical inference of the three-parameter inverse power Lomax distribution under the progressive first failure sample. Some other related studies on inverse distribution can be found in Nassar and Abo-Kasem [19], Lee and Cho [20], Xu and Cui [21] and Rashad et al. [22].

In 2019, a new three-parameter lifetime distribution named the inverse power Lomax (IPL) distribution was introduced by Hassan and Abd-Allah [23]. The probability density function (PDF) f(⋅), cumulative distribution function (CDF) F(⋅) of the IPL distribution are given, respectively, by

(1) $$f(t;\alpha ,\beta ,\lambda )=\alpha \beta \lambda^{-1}t_{}^{-(\beta +1)}{\left(1+\lambda^{-1}t_{}^{-\beta }\right)}^{-\alpha -1},t>0,$$

(2) $$F(t;\alpha ,\beta ,\lambda )={\left(1+\lambda^{-1}t_{}^{-\beta }\right)}^{-\alpha },t>0,$$

where α>0,β>0 are shape parameters, and λ>0 is scale parameter. The IPL is very flexible in analyzing situations with a realized non-monotonic failure rate. Therefore, the IPL model can be used for several practical data modeling and analysis, see Ref. [23]. In order to facilitate engineering applications, Ref. [23] studied some statistical properties for the IPL distribution. The MLEs of the model parameters are obtained based on conventional Type I and Type II censored samples. However, they did not discuss the PFFC scheme. The PFFC scheme is more widely used in survival analysis and the life test.

Since the IPL distribution contains three unknown parameters, it is more complicated to estimate the unknown parameters under progressive censoring. So, to date, there has been no published work on statistical inference for IPL distribution under the PFFC scheme. The main aim of this paper is to focus on the classical and Bayesian inference for IPL distribution under the PFFC scheme.

The rest of this paper is organized as follows: In Section 2, the MLEs and asymptotic confidence intervals of the unknown parameters are derived. Based on Tierney–Kadane’s approximation method, Bayesian estimates of the parameters under squared error loss and generalized entropy loss function are obtained in Section 3. In addition, the highest posterior density (HPD) credible intervals of the parameters are constructed by using the importance sampling method. In Section 4, Monte Carlo simulations are carried out to investigate the performances of different point estimates and interval estimates. In Section 5, a real data set has been analyzed for illustrative purposes. The conclusions are given in Section 6.

## 2. Maximum Likelihood Estimation

In this section, the MLEs of the parameters for the IPL distribution will be discussed under the PFFC. Let $X_{i}=X_{i:m;n},i=1,2,\cdots ,m$ be the progressive first-failure censored order statistics from the IPL distribution with the censored scheme $\underset{˜}{R}=(R_{1},R_{2},\ldots ,R_{m})$. Then, using Equations (1) and (2), the likelihood function is given by

(3) $$\begin{array}{c} L(\alpha ,\beta ,\lambda |\vec{x})=Ck^{m}\prod_{i=1}^{m}f(x_{i};\beta ,\lambda ){\left[1-F(x_{i};\alpha ,\beta ,\lambda )\right]}^{k(R_{i}^{}+1)-1}\\ =Ck^{m}\alpha^{m}\beta^{m}\lambda^{-m}\prod_{i=1}^{m}x_{i}^{-\beta -1}\xi_{i}^{-\alpha -1}{\left(1-\xi_{i}^{-\alpha }\right)}^{k(R_{i}^{}+1)-1},\;0<x_{1}<x_{2}<\ldots <x_{m}, \end{array}$$

where $C=n(n-R_{1}-1)\cdots (n-m+1-R_{1}-\cdots R_{m-1})$, $\vec{x}=(x_{1},x_{2},\cdots ,x_{m})$ and $\xi_{i}^{}=(1+\lambda^{-1}x_{i}^{-\beta })$. The log-likelihood function is given by

(4) $$\ln L(\alpha ,\beta ,\lambda |\vec{x})\propto m\ln\alpha \beta -m\ln\lambda -(\beta +1)\sum_{i=1}^{m}\ln x_{i}-(\alpha +1)\sum_{i=1}^{m}\ln\xi_{i}+\sum_{i=1}^{m}[k(R_{i}+1)-1]\ln(1-\xi_{i}^{-\alpha }).$$

Let $l(\alpha ,\beta ,\lambda |\vec{x})=\ln L(\vec{x};\alpha ,\beta ,\lambda )$. By taking the first partial derivative of log-likelihood function with regard to α,β and λ and equating them to zero, the following results can be obtained.

(5) $$\frac{\partial l}{\partial \alpha }=\frac{m}{\alpha }-\sum_{i=1}^{m}\ln\xi_{i}+\sum_{i=1}^{m}[k(R_{i}+1)-1]\frac{\ln\xi_{i}}{\xi_{i}^{\alpha }-1}=0,$$

(6) $$\frac{\partial l}{\partial \beta }=\frac{m}{\beta }-\sum_{i=1}^{m}\ln x_{i}+\frac{\alpha +1}{\lambda }\sum_{i=1}^{m}\frac{x_{i}^{-\beta }\ln x_{i}}{\xi_{i}}-\sum_{i=1}^{m}[k(R_{i}+1)-1]\frac{\alpha x_{i}^{-\beta }\ln x_{i}}{\lambda (\xi_{i}^{\alpha +1}-\xi_{i})}=0,$$

(7) $$\frac{\partial l}{\partial \lambda }=-\frac{m}{\lambda }+\frac{\alpha +1}{\lambda^{2}}\sum_{i=1}^{m}\frac{x_{i}^{-\beta }}{\xi_{i}}-\sum_{i=1}^{m}[k(R_{i}+1)-1]\frac{\alpha x_{i}^{-\beta }}{\lambda^{2}(\xi_{i}^{\alpha +1}-\xi_{i})}=0.$$

The MLEs of α,β and λ can be obtained by solving the Equations (5)–(7), but these equations do not yield an analytical solution. Therefore, we use the Newton–Raphson iteration method to obtain the MLEs of the parameters. For this purpose, we first compute the quantity of interest $I_{ij},\;i,j=1,2,3$. Here,

(8) $$I_{11}=\frac{\partial^{2}l}{\partial \alpha^{2}}=-\frac{m}{\alpha^{2}}-\sum_{i=1}^{m}[k(R_{i}+1)-1]\frac{\xi_{i}^{\alpha }{\left(\ln\xi_{i}\right)}^{2}}{{\left(\xi_{i}^{\alpha }-1\right)}^{2}},$$

(9) $$I_{12}=I_{21}=\frac{\partial^{2}l}{\partial \alpha \partial \beta }=-\sum_{i=1}^{m}\frac{x_{i}^{-\beta }\ln x_{i}}{\lambda \xi_{i}}+\sum_{i=1}^{m}\left[k(R_{i}+1)-1][\frac{x_{i}^{-\beta }\ln x_{i}}{\lambda \xi_{i}{\left(\xi_{i}^{\alpha }-1\right)}^{2}}-\frac{\alpha \xi_{i}^{\alpha -1}x_{i}^{-\beta }\ln\xi_{i}\ln x_{i}}{\lambda (\xi_{i}^{\alpha +1}-\xi_{i})}\right]\;,$$

(10) $$I_{13}=I_{31}=\frac{\partial^{2}l}{\partial \alpha \partial \lambda }=\sum_{i=1}^{m}\frac{x_{i}^{-\beta }}{\lambda^{2}\xi_{i}}-\sum_{i=1}^{m}\left[k(R_{i}+1)-1][\frac{x_{i}^{-\beta }(\xi_{i}^{\alpha }-1)-\alpha \xi_{i}^{\alpha }x_{i}^{-\beta }\ln\xi_{i}}{\lambda^{2}\xi_{i}{\left(\xi_{i}^{\alpha }-1\right)}^{2}}\right],$$

(11) $$\begin{array}{c} I_{22}=\frac{\partial^{2}l}{\partial \beta^{2}}=-\frac{m}{\beta^{2}}-\frac{\alpha +1}{\lambda }\sum_{i=1}^{m}\frac{1}{\lambda \xi_{i}^{2}}[\lambda x_{i}^{-\beta }\xi_{i}{\left(\ln x_{i}\right)}^{2}-x_{i}^{-2\beta }{\left(\ln x_{i}\right)}^{2}]+\sum_{i=1}^{m}[k(R_{i}+1)-1]\frac{1}{\lambda^{2}{\left(\xi_{i}^{\alpha +1}-\xi_{i}\right)}^{2}}\\ \times [\lambda \alpha x_{i}^{-\beta }{\left(\ln x_{i}\right)}^{2}(\xi_{i}^{\alpha +1}-\xi_{i})-\alpha (\alpha +1)\xi_{i}^{\alpha }x_{i}^{-2\beta }{\left(\ln x_{i}\right)}^{2}+\alpha x_{i}^{-2\beta }{\left(\ln x_{i}\right)}^{2}], \end{array}$$

(12) $$\begin{array}{c} I_{23}=I_{32}=\frac{\partial^{2}l}{\partial \beta \partial \lambda }=\frac{\alpha +1}{\lambda^{2}}\sum_{i=1}^{m}\frac{1}{\xi_{i}^{2}}[-x_{i}^{-\beta }\xi_{i}\ln x_{i}+\lambda^{-1}x_{i}^{-2\beta }\ln x_{i}]+\sum_{i=1}^{m}[k(R_{i}+1)-1]\frac{1}{\lambda^{4}{\left(\xi_{i}^{\alpha +1}-\xi_{i}\right)}^{2}}\\ \times [\alpha \lambda^{2}x_{i}^{-\beta }\ln x_{i}(\xi_{i}^{\alpha +1}-\xi_{i})-\lambda \alpha (\alpha +1)\xi_{i}^{\alpha }x_{i}^{-2\beta }\ln x_{i}-\lambda^{-1}\alpha x_{i}^{-2\beta }\ln x_{i}] \end{array}$$

(13) $$\begin{array}{c} I_{33}=\frac{\partial^{2}l}{\partial \lambda^{2}}=\frac{m}{\lambda^{2}}-\frac{2(\alpha +1)}{\lambda^{3}}\sum_{i=1}^{m}\frac{x_{i}^{-\beta }}{\xi_{i}^{}}+\frac{\alpha +1}{\lambda^{4}}\sum_{i=1}^{m}x_{i}^{-2\beta }\xi_{i}^{-2}\\ +\sum_{i=1}^{m}[k(R_{i}+1)-1][\frac{2\alpha x_{i}^{-\beta }}{\lambda^{3}(\xi_{i}^{\alpha +1}-\xi_{i})}-\frac{\alpha (\alpha +1)x_{i}^{-2\beta }\xi_{i}^{\alpha }}{\lambda^{4}{\left(\xi_{i}^{\alpha +1}-\xi_{i}\right)}^{2}}+\frac{\alpha x_{i}^{-2\beta }}{\lambda^{4}{\left(\xi_{i}^{\alpha +1}-\xi_{i}\right)}^{2}}]. \end{array}$$

The Newton–Raphson iteration method can be implemented according to the following steps:

Step 1: Give the initial values of θ=(α,β,λ), say $\theta^{\left(0\right)}=(\alpha^{\left(0\right)},\beta^{\left(0\right)},\lambda^{\left(0\right)})$.

Step 2: In the *M*-th iteration, calculate $\left(\frac{\partial l}{\partial \alpha },\frac{\partial l}{\partial \beta },\frac{\partial l}{\partial \lambda }\right)|{}_{\theta =\theta^{\left(M\right)}}$ and matrix $I(\theta^{\left(M\right)})$. Here, $I(\theta^{\left(M\right)})=\left[\begin{array}{ccc} -I_{11} & -I_{12} & -I_{13}\\ -I_{21} & -I_{22} & -I_{23}\\ -I_{31} & -I_{32} & -I_{33} \end{array}\right]|{}_{\theta =\theta^{\left(M\right)}}$, $\theta^{\left(M\right)}=(\alpha^{\left(M\right)},\beta^{\left(M\right)},\lambda^{\left(M\right)})$, $I_{ij},\;i,j=1,2,3$ are given by Equations (9)–(14).

Step 3: Update ${\left(\alpha ,\beta ,\lambda \right)}^{T}$ by

(14) $${\left(\alpha^{\left(M+1\right)},\beta^{\left(M+1\right)},\lambda^{\left(M+1\right)}\right)}^{T}={\left(\alpha^{\left(M\right)},\beta^{\left(M\right)},\lambda^{\left(M\right)}\right)}^{T}+I^{-1}(\theta^{\left(M\right)})\times {\left(\frac{\partial l}{\partial \alpha },\frac{\partial l}{\partial \beta },\frac{\partial l}{\partial \lambda }\right)}^{T}|{}_{\theta =\theta^{\left(M\right)}}$$

here, ${\left(\alpha ,\beta ,\lambda \right)}^{T}$ is the transpose of vector (α,β,λ), and $I^{-1}(\theta^{\left(M\right)})$ represents the inverse of matrix $I(\theta^{\left(M\right)})$.

Step 4: Setting M=M+1, the MLEs of θ=(α,β,λ), say $\hat{\theta }=(\hat{\alpha },\hat{\beta },\hat{\lambda })$ can be obtained by repeating steps 2–3 until $\left|{\left(\alpha^{\left(M+1\right)},\beta^{\left(M+1\right)},\lambda^{\left(M+1\right)}\right)}^{T}-{\left(\alpha^{\left(M\right)},\beta^{\left(M\right)},\lambda^{\left(M\right)}\right)}^{T}\right|<\varepsilon$, where ε is a threshold value and fixed in advance.

### Asymptotic Confidence Interval

In this subsection, the asymptotic confidence intervals (ACIs) of the unknown parameters of the IPL distribution are derived. Based on regularity conditions, the MLEs $\left(\hat{\alpha },\hat{\beta },\hat{\lambda }\right)$ are approximately normal distribution with the mean (α,β,λ) and the covariance matrix $\;I^{-1}(\alpha ,\beta ,\lambda )$. In practice, we usually estimate $\;I^{-1}(\alpha ,\beta ,\lambda )$ by $\;I^{-1}(\hat{\alpha },\hat{\beta },\hat{\lambda })$. A simpler and equally valid procedure is to use the approximation $\left(\hat{\alpha },\hat{\beta },\hat{\lambda })∼N((\alpha ,\beta ,\lambda ),\;I^{-1}(\hat{\alpha },\hat{\beta },\hat{\lambda })\right)$, where $\;I^{-1}(\hat{\alpha },\hat{\beta },\hat{\lambda })$ denotes the inverse of observed fisher information matrix $I(\hat{\alpha },\hat{\beta },\hat{\lambda })$ and

(15) $$I(\hat{\alpha },\hat{\beta },\hat{\lambda })={\left[\begin{array}{ccc} -I_{11} & -I_{12} & -I_{13}\\ -I_{21} & -I_{22} & -I_{23}\\ -I_{31} & -I_{32} & -I_{33} \end{array}\right]}_{\left(\alpha ,\beta ,\lambda )=(\hat{\alpha },\hat{\beta },\hat{\lambda }\right)}^{}.$$

Here, $I_{ij},i,j=1,2,3$ can be calculated by Equations (8)–(13), respectively. Thus, the approximate 100(1−γ)% two-sided CIs for parameters α,β,λ are, respectively, given by

(16) $$\left(\hat{\alpha }\pm z_{\gamma /2}\sqrt{\;\hat{V}\mathrm{ar}(\hat{\alpha })}\right),\left(\hat{\beta }\pm z_{\gamma /2}\sqrt{\;\hat{V}\mathrm{ar}(\hat{\beta })}\right),\left(\hat{\lambda }\pm z_{\gamma /2}\sqrt{\;\hat{V}\mathrm{ar}(\hat{\lambda })}\right).$$

Here, $\;\hat{V}\mathrm{ar}(\hat{\alpha })$, $\;\hat{V}\mathrm{ar}(\hat{\beta })$ and $\;\hat{V}\mathrm{ar}(\hat{\lambda })$ are diagonal elements of the observed variance–covariance matrix $\;I^{-1}(\hat{\alpha },\hat{\beta },\hat{\lambda })$, and $z_{\gamma /2}$ is the upper γ/2-th percentile of the standard normal distribution.

## 3. Bayesian Estimation

In this section, we discuss the Bayesian estimates and corresponding credible intervals of the unknown parameters for the IPL distribution. In order to select the best decision in the decision theory, an appropriate loss function must be specified. Here, we consider both the symmetric and asymmetric loss functions. A very well-known symmetric loss function is the square error loss (SEL). The most commonly used asymmetric loss function is the generalized entropy loss (GEL) function. The SEL and GEL function are, respectively, defined by

$$\begin{array}{c} L_{1}(\theta ,\hat{\theta })=(\hat{\theta }-\theta {)}^{2},\\ L_{2}(\theta ,\hat{\theta })\propto {\left(\frac{\hat{\theta }}{\theta }\right)}^{q}-q\ln\left(\frac{\hat{\theta }}{\theta }\right)-1,\;q\neq 0. \end{array}$$

Here, $\hat{\theta }$ is an estimation of θ, and the constant q denotes how much influence that an error will have. When q<0, negative errors affect the consequences more seriously. When q>0, positive errors cause more serious consequences than negative ones.

Under the SEL and GEL function, the Bayesian estimator of θ are, respectively, given by

(17) $${\hat{\theta }}_{BS}=E[\theta |\vec{x}]$$

(18) $${\hat{\theta }}_{BG}={\left[E(\theta^{-q}|\vec{x})\right]}^{-1/q},\begin{array}{cc} & q\neq 0 \end{array}.$$

The Bayesian analysis requires the choice of appropriate priors for the unknown parameters in addition to the experimental data. Arnold and Press [24] correctly pointed out that there is no clear cut way how to choose prior. Now, we assume the following independent gamma priors for the parameters α,β and λ as

$$\begin{array}{l} \pi_{1}(\alpha )\propto \alpha^{a_{1}-1}\exp(-b_{1}\alpha )\;,\;\alpha >0,a_{1},b_{1}>0,\\ \pi_{2}(\beta )\propto \beta^{a_{2}-1}\exp(-b_{2}\beta )\;,\;\beta >0,a_{2},b_{2}>0,\\ \pi_{3}(\lambda )\propto \lambda^{a_{3}-1}\exp(-b_{3}\lambda ),\;\lambda >0,a_{3},b_{3}>0. \end{array}$$

therefore, the joint prior distribution of α,β and λ is given by

(19) $$\pi (\alpha ,\beta ,\lambda )\propto \alpha^{a_{1}-1}\beta^{a_{2}-1}\lambda^{a_{3}-1}\exp(-b_{1}\alpha -b_{2}\beta -b_{3}\lambda ),\;\;\alpha ,\beta ,\lambda >0,a_{i},b_{i},c_{i}>0,i=1,2,3.$$

The assumption of independent gamma priors is reasonable [10]. The class of the gamma prior distributions is quite flexible as it can model a variety of prior information. It should be noted that the non-informative priors on the parameters are the special cases of independent gamma priors and can be achieved by approaching hyper-parameters to zero [10].

Based on Equations (3) and (18), the joint posterior distribution of the parameters α,β and λ can be written as:

(20) $$\pi^{*}(\alpha ,\beta ,\lambda |\vec{x})=\frac{\pi (\alpha ,\beta ,\lambda )L(\vec{x};\alpha ,\beta ,\lambda )}{\int_{0}^{\infty }\int_{0}^{\infty }\int_{0}^{\infty }\pi (\alpha ,\beta ,\lambda )L(\vec{x};\alpha ,\beta ,\lambda )d\alpha d\beta d\lambda }\;\begin{array}{l} \propto \pi (\alpha ,\beta ,\lambda )L(\vec{x};\alpha ,\beta ,\lambda )\\ \propto \alpha^{m+a_{1}-1}\beta^{m+a_{2}-1}\lambda^{m\alpha +a_{3}-1}\exp(-\alpha b_{1}-\beta b_{2}-b_{3}\lambda )\prod_{i=1}^{m}x_{i}^{-\beta -1}\prod_{i=1}^{m}{\left(\lambda +x_{i}^{-\beta }\right)}^{-\alpha -1}\prod_{i=1}^{m}{\left(1-\xi_{i}^{-\alpha }\right)}^{k(R_{i}^{}+1)-1}. \end{array}$$

Let g=g(α,β,λ) be a function of α,β and λ, then the posterior mean of g is given by

(21) $$E[g(\alpha ,\beta ,\lambda )|\vec{x}]=\frac{\int_{0}^{\infty }\int_{0}^{\infty }\int_{0}^{\infty }g(\alpha ,\beta ,\lambda )\pi (\alpha ,\beta ,\lambda )L(\alpha ,\beta ,\lambda |\vec{x})d\alpha d\beta d\lambda }{\int_{0}^{\infty }\int_{0}^{\infty }\int_{0}^{\infty }\pi (\alpha ,\beta ,\lambda )L(\alpha ,\beta ,\lambda |\vec{x})d\alpha d\beta d\lambda }.$$

From Equation (20), we observe that the posterior mean of g(α,β,λ) is in the form of ratio of two integrals for which a closed-form solution is not available [10]. Therefore, we use Tierney–Kadane’s approximation method to obtain the approximate solution of Equation (20).

### 3.1. Tierney–Kadane’s Approximation Method

Tierney and Kadane [25] proposed an alternative method to approximate such a ratio of integrals to derive the Bayesian estimates of unknown parameters. In this subsection, we present the approximate Bayesian estimates of α,β and λ under the SEL and GEL function using Tierney–Kadane’s (T–K) method. Although Lindley’s approximation [26] plays an important role in the Bayesian analysis, this approximation requires the evaluation of third derivatives of the log-likelihood function, which is very tedious in some situations, such as the present one. Moreover, Lindley’s approximation has an error of order $O(n^{-1})$, whereas the T–K approximation has an error of order $O(n^{-2})$.

To apply the T–K approximation method, we set

$$Q(\alpha ,\beta ,\lambda )=\frac{1}{n}[l(\alpha ,\beta ,\lambda |\vec{x})+\ln\pi (\alpha ,\beta ,\lambda )]$$

and

$$Q^{*}(\alpha ,\beta ,\lambda )=Q(\alpha ,\beta ,\lambda )+\frac{1}{n}\ln g(\alpha ,\beta ,\lambda ),$$

where $l(\alpha ,\beta ,\lambda |\vec{x})$ is log-likelihood function. According to the T–K method, the approximation of the posterior mean of g(α,β,λ) is given by

$$E[g(\alpha ,\beta ,\lambda )|\vec{x}]=\frac{\int_{0}^{\infty }\int_{0}^{\infty }\int_{0}^{\infty }\exp\{n\;Q^{*}(\alpha ,\beta ,\lambda )\}d\alpha d\beta d\lambda }{\int_{0}^{\infty }\int_{0}^{\infty }\int_{0}^{\infty }\exp\{n\;Q(\alpha ,\beta ,\lambda )\}d\alpha d\beta d\lambda }$$

Using the T–K approximation method, the approximation of the posterior mean of g(α,β,λ) can be given as

(22) $$\hat{E}[g(\alpha ,\beta ,\lambda )|\vec{x}]=\sqrt{\frac{\det\Sigma^{*}}{\det\Sigma }}\exp\{nQ^{*}({\hat{\alpha }}_{Q*},{\hat{\beta }}_{Q*},{\hat{\lambda }}_{Q*})-nQ({\hat{\alpha }}_{Q},{\hat{\beta }}_{Q},{\hat{\lambda }}_{Q})\}.$$

Here, $\left({\hat{\alpha }}_{Q*},{\hat{\beta }}_{Q*},{\hat{\lambda }}_{Q*}\right)$ and $\left({\hat{\alpha }}_{Q},{\hat{\beta }}_{Q},{\hat{\lambda }}_{Q}\right)$ maximize $Q^{*}(\alpha ,\beta ,\lambda )$ and Q(α,β,λ), respectively. $\Sigma^{*}$ and Σ are the inverse of negative Hessians of $Q^{*}(\alpha ,\beta ,\lambda )$ and Q(α,β,λ) at $\left({\hat{\alpha }}_{Q*},{\hat{\beta }}_{Q*},{\hat{\lambda }}_{Q*}\right)$ and $\left({\hat{\alpha }}_{Q},{\hat{\beta }}_{Q},{\hat{\lambda }}_{Q}\right)$, respectively. For the IPL distribution, we have

(23) $$\begin{array}{c} Q(\alpha ,\beta ,\lambda )=\frac{1}{n}{\{\mathrm{lnCk}}^{m}+m\ln\alpha \beta -m\ln\lambda -(\beta +1)\sum_{i=1}^{m}\ln x_{i}-(\alpha +1)\sum_{i=1}^{m}\ln\xi_{i}+\sum_{i=1}^{m}[k(R_{i}+1)-1]\ln(1-\xi_{i}^{-\alpha })\\ +(a_{1}-1)\ln\alpha -b_{1}\alpha +(a_{2}-1)\ln\beta -b_{2}\beta +\;(a_{3}-1)\ln\lambda -b_{3}\lambda \},\;\alpha ,\beta ,\lambda >0,a_{i},b_{i},c_{i}>0,i=1,2,3. \end{array}$$

Then, $\left({\hat{\alpha }}_{Q},{\hat{\beta }}_{Q},{\hat{\lambda }}_{Q}\right)$ are computed by solving the following non-linear equations.

(24) $$\frac{\partial Q}{\partial \alpha }=\frac{1}{n}\{\frac{m+a_{1}-1}{\alpha }-\sum_{i=1}^{m}\ln\xi_{i}+\sum_{i=1}^{m}[k(R_{i}+1)-1]\frac{\ln\xi_{i}}{\xi_{i}^{\alpha }-1}-b_{1}\}=0,$$

(25) $$\frac{\partial Q}{\partial \beta }=\frac{1}{n}\{\frac{m+a_{2}-1}{\beta }-\sum_{i=1}^{m}\ln x_{i}+\frac{\alpha +1}{\lambda }\sum_{i=1}^{m}\frac{x_{i}^{-\beta }\ln x_{i}}{\xi_{i}}-\sum_{i=1}^{m}[k(R_{i}+1)-1]\frac{\alpha x_{i}^{-\beta }\ln x_{i}}{\lambda (\xi_{i}^{\alpha +1}-\xi_{i})}-b_{2}\}=0\;,$$

(26) $$\frac{\partial Q}{\partial \lambda }=\frac{1}{n}\{\frac{a_{3}-m-1}{\lambda }+\frac{\alpha +1}{\lambda^{2}}\sum_{i=1}^{m}\frac{x_{i}^{-\beta }}{\xi_{i}}-\sum_{i=1}^{m}[k(R_{i}+1)-1]\frac{\alpha x_{i}^{-\beta }}{\lambda^{2}(\xi_{i}^{\alpha +1}-\xi_{i})}-b_{3}\}=0.\;$$

We obtain the Σ from

$$\Sigma ={\left[\begin{array}{ccc} Q_{11} & Q_{12} & Q_{13}\\ Q_{21} & Q_{22} & Q_{23}\\ Q_{31} & Q_{32} & Q_{33} \end{array}\right]}_{\left({\hat{\alpha }}_{Q},{\hat{\beta }}_{Q},{\hat{\lambda }}_{Q}\right)}^{-1},$$

where

$$\begin{array}{c} Q_{11}=-\frac{\partial^{2}Q}{\partial \alpha^{2}}=\frac{1}{n}\{\frac{m+a_{1}-1}{\alpha^{2}}+\sum_{i=1}^{m}[k(R_{i}+1)-1]\frac{\xi_{i}^{\alpha }{\left(\ln\xi_{i}\right)}^{2}}{{\left(\xi_{i}^{\alpha }-1\right)}^{2}}\},\\ Q_{12}=Q_{21}=-\frac{\partial^{2}Q}{\partial \alpha \partial \beta }=\frac{1}{n}\{\sum_{i=1}^{m}\frac{x_{i}^{-\beta }\ln x_{i}}{\lambda \xi_{i}}-\sum_{i=1}^{m}\left[k(R_{i}+1)-1][\frac{x_{i}^{-\beta }\ln x_{i}}{\lambda \xi_{i}{\left(\xi_{i}^{\alpha }-1\right)}^{2}}-\frac{\alpha \xi_{i}^{\alpha -1}x_{i}^{-\beta }\ln\xi_{i}\ln x_{i}}{\lambda (\xi_{i}^{\alpha +1}-\xi_{i})}\right]\}\\ Q_{13}=Q_{31}=-\frac{\partial^{2}Q}{\partial \alpha \partial \lambda }=\frac{1}{n}\{-\sum_{i=1}^{m}\frac{x_{i}^{-\beta }}{\lambda^{2}\xi_{i}}+\sum_{i=1}^{m}\left[k(R_{i}+1)-1][\frac{x_{i}^{-\beta }(\xi_{i}^{\alpha }-1)-\alpha \xi_{i}^{\alpha }x_{i}^{-\beta }\ln\xi_{i}}{\lambda^{2}\xi_{i}{\left(\xi_{i}^{\alpha }-1\right)}^{2}}]\right\},\\ Q_{22}=-\frac{\partial^{2}Q}{\partial \beta^{2}}=\frac{1}{n}\{\frac{m+a_{2}-1}{\beta^{2}}+\frac{\alpha +1}{\lambda }\sum_{i=1}^{m}\frac{1}{\lambda \xi_{i}^{2}}[\lambda x_{i}^{-\beta }\xi_{i}{\left(\ln x_{i}\right)}^{2}-x_{i}^{-2\beta }{\left(\ln x_{i}\right)}^{2}]-\sum_{i=1}^{m}[k(R_{i}+1)-1]\frac{1}{\lambda^{2}{\left(\xi_{i}^{\alpha +1}-\xi_{i}\right)}^{2}}\\ \times [\lambda \alpha x_{i}^{-\beta }{\left(\ln x_{i}\right)}^{2}(\xi_{i}^{\alpha +1}-\xi_{i})-\alpha (\alpha +1)\xi_{i}^{\alpha }x_{i}^{-2\beta }{\left(\ln x_{i}\right)}^{2}+\alpha x_{i}^{-2\beta }{\left(\ln x_{i}\right)}^{2}]\},\\ Q_{23}=Q_{32}=-\frac{\partial^{2}Q}{\partial \beta \partial \lambda }=\frac{1}{n}\{-\frac{\alpha +1}{\lambda^{2}}\sum_{i=1}^{m}\frac{1}{\xi_{i}^{2}}[-x_{i}^{-\beta }\xi_{i}\ln x_{i}+\lambda^{-1}x_{i}^{-2\beta }\ln x_{i}]-\sum_{i=1}^{m}[k(R_{i}+1)-1]\frac{1}{\lambda^{4}{\left(\xi_{i}^{\alpha +1}-\xi_{i}\right)}^{2}}\\ \times [\alpha \lambda^{2}x_{i}^{-\beta }\ln x_{i}(\xi_{i}^{\alpha +1}-\xi_{i})-\lambda \alpha (\alpha +1)\xi_{i}^{\alpha }x_{i}^{-2\beta }\ln x_{i}-\lambda^{-1}\alpha x_{i}^{-2\beta }\ln x_{i}]\}\\ Q_{33}=-\frac{\partial^{2}Q}{\partial \lambda^{2}}=\frac{1}{n}\{\frac{a_{3}-m-1}{\lambda^{2}}+\frac{2(\alpha +1)}{\lambda^{3}}\sum_{i=1}^{m}\frac{x_{i}^{-\beta }}{\xi_{i}^{}}-\frac{\alpha +1}{\lambda^{4}}\sum_{i=1}^{m}x_{i}^{-2\beta }\xi_{i}^{-2}\\ -\sum_{i=1}^{m}[k(R_{i}+1)-1][\frac{2\alpha x_{i}^{-\beta }}{\lambda^{3}(\xi_{i}^{\alpha +1}-\xi_{i})}-\frac{\alpha (\alpha +1)x_{i}^{-2\beta }\xi_{i}^{\alpha }}{\lambda^{4}{\left(\xi_{i}^{\alpha +1}-\xi_{i}\right)}^{2}}+\frac{\alpha x_{i}^{-2\beta }}{\lambda^{4}{\left(\xi_{i}^{\alpha +1}-\xi_{i}\right)}^{2}}]\}. \end{array}$$

Based on the T–K approximation method, we can derive the Bayesian estimates of the parameters α,β and λ under the different loss functions.

(I) Squared error loss function

In order to compute the Bayesian estimator of unknown parameters under the squared error loss function (SELF), we take g(α,β,λ)=α, and accordingly, the function $Q_{\alpha }^{*}(\alpha ,\beta ,\lambda )$ becomes

$$Q_{\alpha }^{*}(\alpha ,\beta ,\lambda )=Q(\alpha ,\beta ,\lambda )+\frac{1}{n}\ln\alpha$$

The MLE $\left({\hat{\alpha }}_{Q*},{\hat{\beta }}_{Q*},{\hat{\lambda }}_{Q*}\right)$ of (α,β,λ) can be obtained by solving the following system of the equations.

$$\frac{\partial Q_{\alpha }^{*}}{\partial \alpha }=\frac{\partial Q}{\partial \alpha }+\frac{1}{n\alpha }=0,\;\frac{\partial Q_{\alpha }^{*}}{\partial \beta }=\frac{\partial Q}{\partial \beta }=0,\;\frac{\partial Q_{\alpha }^{*}}{\partial \lambda }=\frac{\partial Q}{\partial \lambda }=0$$

Thus, $\Sigma_{\alpha }^{*}$ can be calculated by

$$\Sigma_{\alpha }^{*}={\left[\begin{array}{ccc} Q_{\alpha 11}^{*} & Q_{\alpha 12}^{*} & Q_{\alpha 13}^{*}\\ Q_{\alpha 21}^{*} & Q_{\alpha 22}^{*} & Q_{\alpha 23}^{*}\\ Q_{\alpha 31}^{*} & Q_{\alpha 32}^{*} & Q_{\alpha 33}^{*} \end{array}\right]}_{\left({\hat{\alpha }}_{Q*},{\hat{\beta }}_{Q*},{\hat{\lambda }}_{Q*}\right)}^{-1}$$

where

$$\begin{array}{c} Q_{\alpha 11}^{*}=-\frac{\partial^{2}Q_{\alpha }^{*}}{\partial \alpha^{2}}=-\frac{\partial^{2}Q}{\partial \alpha^{2}}+\frac{1}{n\alpha^{2}},\;Q_{\alpha 12}^{*}=Q_{\alpha 21}^{*}=-\frac{\partial^{2}Q_{\alpha }^{*}}{\partial \alpha \partial \beta }=-\frac{\partial^{2}Q}{\partial \alpha \partial \beta },Q_{\alpha 13}^{*}=Q_{\alpha 31}^{*}=-\frac{\partial^{2}Q_{\alpha }^{*}}{\partial \alpha \partial \lambda }=-\frac{\partial^{2}Q}{\partial \alpha \partial \lambda },\\ Q_{\alpha 22}^{*}=-\frac{\partial^{2}Q_{\alpha }^{*}}{\partial \beta^{2}}=-\frac{\partial^{2}Q}{\partial \beta^{2}},Q_{\alpha 23}^{*}=Q_{\alpha 32}^{*}=-\frac{\partial^{2}Q_{\alpha }^{*}}{\partial \beta \partial \lambda }=-\frac{\partial^{2}Q}{\partial \alpha \partial \beta },Q_{\alpha 33}^{*}=-\frac{\partial^{2}Q_{\alpha }^{*}}{\partial \lambda^{2}}=-\frac{\partial^{2}Q}{\partial \lambda^{2}}, \end{array}$$

Under SELF, the Bayesian estimator of α is given by

$${\hat{\alpha }}_{BS}=\sqrt{\frac{\det\Sigma^{*}}{\det\Sigma }}\exp\{nQ_{\alpha }^{*}({\hat{\alpha }}_{Q*},{\hat{\beta }}_{Q*},{\hat{\lambda }}_{Q*})-nQ({\hat{\alpha }}_{Q},{\hat{\beta }}_{Q},{\hat{\lambda }}_{Q})\}.$$

Similarly, the Bayesian estimators of β and λ under SELF are given, respectively, by

$$\begin{array}{c} {\hat{\beta }}_{BS}=\sqrt{\frac{\det\Sigma^{*}}{\det\Sigma }}\exp\{nQ_{\beta }^{*}({\hat{\alpha }}_{Q*},{\hat{\beta }}_{Q*},{\hat{\lambda }}_{Q*})-nQ({\hat{\alpha }}_{Q},{\hat{\beta }}_{Q},{\hat{\lambda }}_{Q})\},\\ {\hat{\lambda }}_{BS}=\sqrt{\frac{\det\Sigma^{*}}{\det\Sigma }}\exp\{nQ_{\lambda }^{*}({\hat{\alpha }}_{Q*},{\hat{\beta }}_{Q*},{\hat{\lambda }}_{Q*})-nQ({\hat{\alpha }}_{Q},{\hat{\beta }}_{Q},{\hat{\lambda }}_{Q})\}. \end{array}$$

(II) General entropy loss function

Firstly, we compute the Bayesian estimator of parameter α. In this case, $g(\alpha ,\beta ,\lambda )=\alpha^{-q}$, then function $Q_{\alpha }^{*}(\alpha ,\beta ,\lambda )$ is given by

$$Q_{\alpha }^{*}(\alpha ,\beta ,\lambda )=Q(\alpha ,\beta ,\lambda )-\frac{q}{n}\ln\alpha$$

By solving the following system of the equations, we obtain the maximum likelihood estimator $\left({\hat{\alpha }}_{Q*},{\hat{\beta }}_{Q*},{\hat{\lambda }}_{Q*}\right)$ of α,β and λ.

$$\frac{\partial Q_{\alpha }^{*}}{\partial \alpha }=\frac{\partial Q}{\partial \alpha }-\frac{q}{n\alpha }=0,\;\frac{\partial Q_{\alpha }^{*}}{\partial \beta }=\frac{\partial Q}{\partial \beta }=0,\;\frac{\partial Q_{\alpha }^{*}}{\partial \lambda }=\frac{\partial Q}{\partial \lambda }=0$$

Thus, $\Sigma_{\alpha }^{*}$ can be calculated by

$$\Sigma_{\alpha }^{*}={\left[\begin{array}{ccc} Q_{\alpha 11}^{*} & Q_{\alpha 12}^{*} & Q_{\alpha 13}^{*}\\ Q_{\alpha 21}^{*} & Q_{\alpha 22}^{*} & Q_{\alpha 23}^{*}\\ Q_{\alpha 31}^{*} & Q_{\alpha 32}^{*} & Q_{\alpha 33}^{*} \end{array}\right]}_{\left({\hat{\alpha }}_{Q*},{\hat{\beta }}_{Q*},{\hat{\lambda }}_{Q*}\right)}^{-1}$$

where

$$\begin{array}{c} Q_{\alpha 11}^{*}=-\frac{\partial^{2}Q_{\alpha }^{*}}{\partial \alpha^{2}}=-\frac{\partial^{2}Q}{\partial \alpha^{2}}-\frac{q}{n\alpha^{2}},\;Q_{\alpha 12}^{*}=Q_{\alpha 21}^{*}=-\frac{\partial^{2}Q_{\alpha }^{*}}{\partial \alpha \partial \beta }=-\frac{\partial^{2}Q}{\partial \alpha \partial \beta },Q_{\alpha 13}^{*}=Q_{\alpha 31}^{*}=-\frac{\partial^{2}Q_{\alpha }^{*}}{\partial \alpha \partial \lambda }=-\frac{\partial^{2}Q}{\partial \alpha \partial \lambda },\\ Q_{\alpha 22}^{*}=-\frac{\partial^{2}Q_{\alpha }^{*}}{\partial \beta^{2}}=-\frac{\partial^{2}Q}{\partial \beta^{2}},Q_{\alpha 23}^{*}=Q_{\alpha 32}^{*}=-\frac{\partial^{2}Q_{\alpha }^{*}}{\partial \beta \partial \lambda }=-\frac{\partial^{2}Q}{\partial \alpha \partial \beta },Q_{\alpha 33}^{*}=-\frac{\partial^{2}Q_{\alpha }^{*}}{\partial \lambda^{2}}=-\frac{\partial^{2}Q}{\partial \lambda^{2}}, \end{array}$$

The Bayesian estimator of α under the general entropy loss function (GELF) is given by

$${\hat{\alpha }}_{BG}={\left\{\sqrt{\frac{\det\Sigma^{*}}{\det\Sigma }}\exp[nQ_{\alpha }^{*}({\hat{\alpha }}_{Q*},{\hat{\beta }}_{Q*},{\hat{\lambda }}_{Q*})-nQ({\hat{\alpha }}_{Q},{\hat{\beta }}_{Q},{\hat{\lambda }}_{Q})]\right\}}^{-1/q}.$$

Similarly, the Bayesian estimators of β and λ under GELF are given by, respectively,

$$\begin{array}{l} {\hat{\beta }}_{BG}={\left\{\sqrt{\frac{\det\Sigma^{*}}{\det\Sigma }}\exp[nQ_{\beta }^{*}({\hat{\alpha }}_{Q*},{\hat{\beta }}_{Q*},{\hat{\lambda }}_{Q*})-nQ({\hat{\alpha }}_{Q},{\hat{\beta }}_{Q},{\hat{\lambda }}_{Q})]\right\}}^{-1/q},\\ {\hat{\lambda }}_{BG}={\left\{\sqrt{\frac{\det\Sigma^{*}}{\det\Sigma }}\exp[nQ_{\lambda }^{*}({\hat{\alpha }}_{Q*},{\hat{\beta }}_{Q*},{\hat{\lambda }}_{Q*})-nQ({\hat{\alpha }}_{Q},{\hat{\beta }}_{Q},{\hat{\lambda }}_{Q})]\right\}}^{-1/q}. \end{array}$$

### 3.2. The Highest Posterior Density Credible Interval

In the previous subsection, we used the T–K approximation method to obtain Bayesian point estimation of unknown parameters. However, this approximation method cannot determine the Bayesian credible intervals of unknown parameters. The importance sampling method is an effective approach to attain the Bayesian credible interval of unknown parameters. Kundu [27] considered Bayesian estimation for the Marshall–Olkin bivariate Weibull distribution, and the Bayesian estimates and associated credible intervals of the unknown parameters were constructed using the importance sampling method. Maurya et al. [28] derived the HPD credible intervals of unknown parameters in a Burr Type XII distribution using the importance sampling method. Sultana et al. [29] considered the estimation of unknown parameters for two-parameter Kumaraswamy distribution with hybrid censored samples. In the subsection, we use the importance sampling method to obtain the HPD credible intervals of unknown parameters of the inverse power Lomax distribution.

Based on the Equation (19), the joint posterior distribution of the parameters α,β and λ can be rewritten as

(27) $$\begin{array}{c} \pi^{*}(\alpha ,\beta ,\lambda |\vec{x})\propto \alpha^{m+a_{1}-1}\beta^{m+a_{2}-1}\lambda^{m\alpha +a_{3}-1}\exp(-\alpha b_{1}-\beta b_{2}-b_{3}\lambda )\prod_{i=1}^{m}x_{i}^{-\beta -1}\prod_{i=1}^{m}{\left(\lambda +x_{i}^{-\beta }\right)}^{-\alpha -1}\prod_{i=1}^{m}{\left(1-\xi_{i}^{-\alpha }\right)}^{k(R_{i}^{}+1)-1}\\ \begin{array}{l} =\alpha^{m+a_{1}-1}\;V_{1}^{m+a_{1}}\exp(-\alpha V_{1})\beta^{m+a_{2}-1}V_{2}^{m+a_{2}}\exp(-\alpha V_{2})\lambda^{m\alpha +a_{3}-1}\exp(-b_{3}\lambda )\\ \times V_{1}^{-(m+a_{1})}V_{2}^{-(m+a_{2})}\prod_{i=1}^{m}{\left(\lambda +x_{i}^{-\beta }\right)}^{-1}\prod_{i=1}^{m}{\left(1-\xi_{i}^{-\alpha }\right)}^{k(R_{i}^{}+1)-1} \end{array}\\ \propto \pi_{1}^{*}(\alpha |\beta ,\lambda ,\vec{x})\pi_{2}^{*}(\beta |\alpha ,\lambda ,\vec{x})\pi_{3}^{*}(\lambda |\alpha ,\beta ,\vec{x})W(\alpha ,\beta ,\lambda |\vec{x}), \end{array}$$

where

$$\begin{array}{l} \pi_{1}^{*}(\alpha |\beta ,\lambda ,\vec{x})\propto \alpha^{m+a_{1}-1}\;V_{1}^{m+a_{1}}\exp(-\alpha V_{1}),\\ \pi_{2}^{*}(\beta |\alpha ,\lambda ,\vec{x})\propto \beta^{m+a_{2}-1}V_{2}^{m+a_{2}}\exp(-\alpha V_{2}),\\ \pi_{3}^{*}(\lambda |\alpha ,\beta ,\vec{x})\propto \lambda^{m\alpha +a_{3}-1}\exp(-b_{3}\lambda ),\\ W(\alpha ,\beta ,\lambda |\vec{x}),\propto V_{1}^{-(m+a_{1})}V_{2}^{-(m+a_{2})}\prod_{i=1}^{m}{\left(\lambda +x_{i}^{-\beta }\right)}^{-1}\prod_{i=1}^{m}{\left(1-\xi_{i}^{-\alpha }\right)}^{k(R_{i}^{}+1)-1}\\ V_{1}=b_{1}+\sum_{i=1}^{m}\ln(\lambda +x_{i}^{-\beta }),\;V_{2}=b_{2}+\sum_{i=1}^{m}\ln x_{i} \end{array}$$

It is observed that $\pi_{1}^{*}(\alpha |\beta ,\lambda ,\vec{x})$ is the PDF of the Gamma distribution $Ga(m+a_{1},V_{1})$, $\pi_{2}^{*}(\beta |\alpha ,\lambda ,\vec{x})$ and $\pi_{3}^{*}(\lambda |\alpha ,\beta ,\vec{x})$ are the PDF of the Gamma distribution $Ga(m+a_{2},V_{2})$ and $Ga(\alpha m+a_{3},b_{3})$, respectively. To obtain the HPD credible intervals for unknown parameters, the importance sampling method is used and the steps as follows.

Step 1: Generate $\alpha_{1}$ from $\pi_{1}^{*}(\alpha |\beta ,\lambda ,\vec{x})$,

Step 2: Generate $\beta_{1}$ from $\pi_{2}^{*}(\beta |\alpha ,\lambda ,\vec{x})$,

Step 3: Generate $\lambda_{1}$ from $\pi_{3}^{*}(\lambda |\alpha ,\beta ,\vec{x})$.

Repeat the steps 1–3 N times, we get $\left(\alpha_{1},\beta_{1},\lambda_{1}),(\alpha_{2},\beta_{2},\lambda_{2}),\cdots ,(\alpha_{N},\beta_{N},\lambda_{N}\right)$.

The 100(1-γ)% Bayesian credible intervals for unknown parameters can be constructed by using the method given in Ref. [27]. We briefly discuss the method below. Let g(α,β,λ) be any function of (α,β,λ). For 0<γ<1, suppose that $g_{\gamma }$ satisfies $P(g(\alpha ,\beta ,\lambda )\leq g_{\gamma }|\vec{x})=\gamma$. Using the sample $(\alpha_{1},\beta_{1},\lambda_{1}),$ $(\alpha_{2},\beta_{2},\lambda_{2}),$ $\cdots ,(\alpha_{N},\beta_{N},\lambda_{N})$, we can calculate $W(\alpha_{i},\beta_{i},\lambda_{i}|\vec{x})$ and $g(\alpha_{i},\beta_{i},\lambda_{i})$. For simplicity, we let $g_{i}=g(\alpha_{i},\beta_{i},\lambda_{i})$ and

$$u_{i}=\frac{W(\alpha_{i},\beta_{i},\lambda_{i}|\vec{x})}{\sum_{i=1}^{N}W(\alpha_{i},\beta_{i},\lambda_{i}|\vec{x})},i=1,2,\cdots ,N.$$

When γ is given, we can attain the estimation of $g_{\gamma }$ and use it to establish the HPD credible intervals for g(α,β,λ).

Rearrange $\left(g_{1},u_{1}),(g_{2},u_{2}),\cdots ,(g_{N},u_{N}\right)$ into $\left(g_{\left(1\right)},u_{\left(1\right)}),(g_{\left(2\right)},u_{\left(2\right)}),\cdots ,(g_{\left(N\right)},u_{\left(N\right)}\right)$, where $g_{\left(1\right)}<g_{\left(2\right)}<\cdots g_{\left(N\right)},$ denote the ordered values of $g_{i},$, and $g_{\left(i\right)}$ is related to $u_{\left(i\right)}$, but $u_{\left(i\right)}$ is not ordered for  i=1,2,⋯,N. The estimator of $g_{\gamma }$ is ${\hat{g}}_{\gamma }=g_{\left(H\gamma \right)},$ where Hγ is an integer satisfying

$$\sum_{i=1}^{H\gamma }u_{\left(i\right)}\leq \gamma <\sum_{i=1}^{H\gamma +1}u_{\left(i\right)}\;$$

Using the above method, a 100(1-γ)% Bayesian credible interval of the function g(α,β,λ) can be given by $({\hat{g}}_{\delta },{\hat{g}}_{\delta +1-\gamma }),$ for $\delta =u_{\left(1\right)},u_{\left(1\right)}+u_{\left(2\right)},\cdots ,\sum_{i=1}^{H_{\left(1-\gamma \right)}}u_{\left(i\right)}.$ Therefore, a 100(1-γ)% HPD credible interval of g(α,β,λ) is given by

$$({\hat{g}}_{\delta^{*}},\;\;{\hat{g}}_{\delta^{*}+1-\gamma }),$$

where $\delta^{*}$ satisfies ${\hat{g}}_{\delta^{*}+1-\gamma }-{\hat{g}}_{\delta^{*}}\leq {\hat{g}}_{\delta +1-\gamma }-{\hat{g}}_{\delta }$ for all δ, and g(α,β,λ) could be α,β,λ, respectively. So, we obtain the HPD credible intervals for unknown parameters of α,β,λ.

## 4. Simulation Study

In this section, we evaluate the performance of different estimates developed in this paper by the Monte Carlo simulation study. For the given true values of parameters α,β,λ and different combinations of $\left(n,m,k,\underset{˜}{R}\right)$, progressive first-failure censored samples are generated from the IPL distribution by modifying the method introduced by Ref. [30]. The following steps provide the specific generation method.

Step 1: Set the initial values of both group size k and censoring scheme $\underset{˜}{R}=(R_{1},R_{2},\ldots ,R_{m})$.
Step 2: Generate m independent observations $Z_{1},Z_{2},\ldots ,Z_{m}$ that obey the uniform distribution U(0,1).
Step 3: Let $\xi_{i}=Z_{i}{}^{1/(i+R_{m}+R_{m-1}+\ldots +R_{m-i+1})}$, i=1,2,⋯,m..
Step 4: Set $U_{i}=1-\xi_{m}\xi_{m-1}\ldots \xi_{m-i+1},i=1,2,\cdots ,m$.
Step 5: For given α,β and λ, using inverse transformation $X_{i}=F^{-1}(U_{i})$, i=1,2,⋯,m, we obtain the PFF censored sample from IPL distribution, where $F^{-1}(\cdot )$ represents the inverse CDF in (2).

In the simulation study, the true values of parameters in the IPL distribution are taken as α=1.5, β=1,λ=0.5. For Bayesian estimates, the means of prior distributions are equal to the true values of the parameters, that is, $a_{1}/b_{1}=\alpha ,\;a_{2}/b_{2}=\beta$, $a_{3}/b_{3}=\lambda$. Therefore, the true values of the hyper-parameters in prior distribution are taken as $\left(a_{1},b_{1})=(1.5,\;1),(a_{2},b_{2})=(1,\;1\right)$, $(a_{3},b_{3})=(1,\;2).$ For the GELF, we set q=−0.5, 0.5,1.0. Two different group sizes k=2,3 are chosen, and two different combinations for n and m say n=30, m=15,20,30; n=50, m=25,30,50 with different $R_{i}$ are determined. For convenience, the different censoring schemes (CS) used in this paper have been represented by short notations, such as (0*4) denotes (0,0,0,0) and ((2,0)*3) denotes (2,0, 2,0, 2,0).

In each case, we compute the MLEs and Bayesian estimates of the unknown parameters. In the Newton iterative algorithm and importance sampling algorithm, we choose the initial values of α,β and λ as $\alpha^{\left(0\right)}=1.4,\beta^{\left(0\right)}=0.9,\lambda^{\left(0\right)}=0.4;$ the value of ε is taken as $10^{-5}$. All Bayesian points and interval estimates are computed under two different loss functions, SELF and GELF, using the the T–K approximation and importance sampling methods, respectively. In addition, we obtain the average length (AL) of 95% asymptotic confidence and HPD credible intervals and corresponding coverage probability (CP) of the parameters based on the simulation. Here, we use N = 2000 for the importance sampling procedure and use M = 2000 simulated samples in each case.

The expected values (EV) and mean square error (MSE) of different estimates are computed. Here, $\mathrm{EV}=M^{-1}\sum \hat{g}(\theta )$ and $MSE=M^{-1}\sum \left[\hat{g}(\theta \right)-g(\theta ){]}^{2}$, where $\hat{g}(\theta )$ is the estimate of g(θ).

Extensive computations are performed using R statistical programming language software. The results of ML and Bayesian point estimates using the Monte Carlo simulation are presented in Table 1, Table 2, Table 3, Table 4 and Table 5. From these tables, the following observations can be made:

1. When n increases but m and k are fixed, the MSEs of MLEs and Bayesian estimates of three parameters decrease. Therefore, we tend to get better estimation results with an increase in sample size.
2. When m increases but n and k are fixed, the MSEs of MLEs and Bayesian estimates decrease. While when k increases but n and m are fixed, the MSEs of all estimates decrease in most of the cases.
3. In the case of Bayesian estimates, there is little difference between the MSEs under SELF and GELF, and the estimation effect of GELF is slightly better than SELF in terms of MSE. While under GELF, there is no significant difference in MSEs among the three modes. The estimation effect seems better when *q* = 1.

Furthermore, the average lengths of 95% asymptotic confidence HPD credible intervals were computed. These results are displayed in Table 6 and Table 7. From the obtained results in Table 6 and Table 7, the following conclusions can be drawn:

1. When *n* increases but *m* and *k* are fixed, the average length of asymptotic confidence and HPD credible intervals narrow down. While the average length of 95% asymptotic confidence and HPD credible intervals narrow down when the group size *k* increases.
2. When *m* increases but n and *k* are fixed, the average length of 95% asymptotic confidence HPD credible intervals narrow down in most of the cases.
3. The HPD credible intervals are better than asymptotic confidence intervals in respect of average length.
4. For the CPs of interval for the unknown parameters, the HPD credible intervals are slightly better than asymptotic confidence intervals in almost all cases.

## 5. Real Data Analysis

In this section, a real data set is considered to illustrate the proposed method. The data set represents the survival times (in days) of 72 guinea pigs infected with virulent tubercle bacilli. This data set was observed and reported by Bjerkedal [31]. The data are listed as follows: 0.1, 0.33, 0.44, 0.56, 0.59, 0.59, 0.72, 0.74, 0.92, 0.93, 0.96, 1, 1, 1.02, 1.05, 1.07, 1.07, 1.08, 1.08, 1.08, 1.09, 1.12, 1.13, 1.15, 1.16, 1.2, 1.21, 1.22, 1.22, 1.24, 1.3, 1.34,1.36, 1.39, 1.44, 1.46, 1.53, 1.59, 1.6, 1.63, 1.63, 1.68, 1.71, 1.72, 1.76, 1.83, 1.95,1.96, 1.97, 2.02, 2.13, 2.15, 2.16, 2.22, 2.3, 2.31, 2.4, 2.45, 2.51, 2.53, 2.54, 2.54, 2.78,2.93, 3.27, 3.42, 3.47, 3.61, 4.02, 4.32, 4.58, 5.55.

The above data set was analyzed by Hassan and Abd-Allah [23] in fitting the IPL distribution (IPLD). The IPLD was compared with Lomax (L), exponentiated Lomax (EL), power Lomax (PL), inverse Weibull (IW), generalized inverse Weibull (GIW) and inverse Lomax (IL) distribution, respectively. The method of maximum likelihood is used to estimate the unknown parameters of the selected models. The following statistics: Akaike information criterion (AIC), the corrected Akaike information criterion (CAIC), Bayesian formation criterion (BIC), the Hannan–Quinn information criterion (HQIC), and Kolmogorov–Smirnov (K–S) statistic was used to compare all the models.

**Figure 1:**
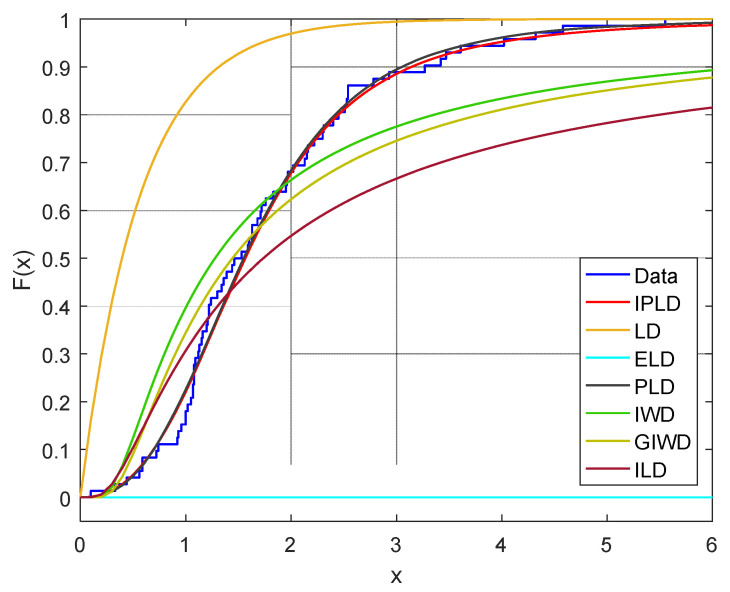
Empirical CDF against CDF of IPLD, LD, ELD, PLD, IWD, GIWD and ILD for the given data set.

In this section, all computations are performed using R statistical programming language software. Table 8 lists the values of MLEs of the parameters, AIC, CAIC, BIC, HQIC and K-S statistic for the considered models. The plots of the estimated CDFs of the fitted distributions are displayed in Figure 1.

From the numerical results in Table 8, it can be seen that the most fitted distribution to these data is IPLD compared to other distributions since the IPLD has the lower statistics. According to the results in Figure 1, it is clear that the IPLD is the most appropriate model for this data set. Therefore, we can perform statistical analysis on this data set.

To analyze this data set under PFF censored samples, we randomly divide the given data into 36 groups with k=2 independent items within each group. Then the following first-failure censored data are obtained: 0.1, 0.44, 0.59, 0.74, 0.93, 1, 1.05, 1.07, 1.08, 1.12, 1.15, 1.2, 1.22, 1.24, 1.4, 1.34, 1.39, 1.46, 1.59, 1.63, 1.68, 1.72, 1.83, 1.97, 2.02, 2.15, 2.22, 2.31, 2.45, 2.53, 2.54, 2.78, 2.93, 3.42, 3.61, 4.02.

Next, we generate progressive first-failure censored samples using three different censoring schemes from the above first-failure censored sample with *m* = 26. The different censoring schemes and the corresponding progressive first-failure censored samples are presented in Table 9. In the different censoring schemes, we calculate the ML and Bayesian estimates of the parameters. For Bayesian estimates, we use non-informative priors as we have no prior information about the parameters. We obtain 95% asymptotic confidence and HPD credible intervals for the parameters. The results of all estimates are listed in Table 10, Table 11 and Table 12.

## 6. Conclusions

In this paper, the statistical inference of the parameters of inverse power Lomax distribution has been studied based on a progressive first-failure censoring sample. Both the classical and Bayesian estimates of the parameters are provided. Since the MLEs of the parameters cannot be obtained in closed form, an iterative procedure has been used. Using the asymptotic normality theory of MLEs, we have developed the approximate confidence intervals of the parameters. The Bayesian estimates are derived by Tierney–Kadane’s approximation method under square error loss and generalized entropy loss functions. Since Tierney–Kadane’s method fails to construct the Bayesian credible intervals, we utilize the importance sampling procedure to obtain the HPD credible intervals of the parameters. A Monte Carlo simulation has been provided to show all the estimation results. Finally, a real data set has been analyzed to illustrate our model. In this paper, although we have used Newton’s iterative method to obtain maximum likelihood estimates of parameters for IPL distribution, other methods such as the gradient and the conjugate gradient methods can also be considered. These methods were proposed by Boumaraf et al. [32], and some good conclusions were obtained. The application of these new methods in parameter statistical inference and reliability analysis will be one of our future research topics.

## Figures and Tables

**Table 1:** MLEs and MSEs of the parameters when α=1.5,β=1,λ=0.5,M=2000.

*k*	*n*	*m*	Censoring Scheme	${\hat{\alpha }}_{ML}$		${\hat{\beta }}_{ML}$		${\hat{\lambda }}_{ML}$	
				EV	MSE	EV	MSE	EV	MSE
2	30	15	(15, 0*14)	1.6392	0.1327	0.8925	0.1084	0.5929	0.1061
			(0*6, 6, 5, 4, 0*6)	1.6342	0.1359	0.8893	0.1106	0.5967	0.1146
			(0*14, 15)	1.6415	0.1391	0.8843	0.1138	0.5974	0.1076
		20	(10, 0 *19)	1.6240	0.1175	0.9188	0.0946	0.5818	0.0917
			(1, 0)*10	1.6392	0.1296	0.8893	0.1084	0.6029	0.1048
			(0*19, 10)	1.6350	0.1371	0.9109	0.1033	0.5821	0.0942
		30	(0*30)	1.5471	0.0721	0.9517	0.0655	0.5519	0.0697
	50	25	(25, 0*24)	1.5861	0.0934	0.9217	0.0886	0.5759	0.0824
			(0*8, 1, 3*8, 0*8)	1.5932	0.0956	0.9159	0.0908	0.5763	0.0831
			(0*24, 25)	1.5916	0.0940	0.9214	0.0894	0.5640	0.0828
		30	(20, 0*29)	1.5735	0.0714	0.9497	0.0796	0.5631	0.0772
			(2, 0, 0)*10	1.5742	0.0756	0.9459	0.0875	0.5515	0.0810
			(0*29, 20)	1.5769	0.0751	0.9353	0.0818	0.5541	0.0780
		50	(0*50)	1.5328	0.0704	0.9738	0.0637	0.5468	0.0638
3	30	15	(15, 0*14)	1.6387	0.1316	0.9162	0.0928	0.5725	0.0955
			(0*6, 6, 5, 4, 0*6)	1.6306	0.1353	0.9101	0.0934	0.5748	0.0994
			(0*14, 15)	1.6354	0.1387	0.9018	0.0954	0.5732	0.1004
		20	(10, 0*19)	1.6271	0.1047	0.9303	0.0832	0.5721	0.0829
			(1, 0)*10	1.6230	0.1212	0.9109	0.0944	05734	0.0904
			(0*19, 10)	1.6245	0.1381	0.9235	0.0946	0.5745	0.0931
		30	(0*30)	1.5466	0.0713	0.9636	0.0647	0.5516	0.0690
	50	25	(25, 0*24)	1.5850	0.0936	0.9323	0.0782	0.5641	0.0784
			(0*8, 1, 3*8, 0*8)	1.5878	0.0965	0.9318	0.0795	0.5315	0.0791
			(0*24,25)	1.5901	0.0972	0.9305	0.0899	0.5419	0.0815
		30	(20, 0*29)	1.5714	0.0732	0.9588	0.0665	0.5501	0.0692
			(2, 0, 0)*10	1.5731	0.0767	0.9585	0.0687	0.5581	0.0711
			(0*29, 20)	1.5762	0.0794	1.0480	0.0856	0.5534	0.0766
		50	(0*50)	1.5218	0.0659	0.9743	0.0632	0.5427	0.0633

**Table 2:** Bayesian estimates and MSEs of the parameters under SELF, when α=1.5,β=1,λ=0.5,M=2000.

*k*	*n*	*m*	Censoring Scheme	${\hat{\alpha }}_{\mathrm{BS}}$		${\hat{\beta }}_{\mathrm{BS}}$		${\hat{\lambda }}_{\mathrm{BS}}$	
				EV	MSE	EV	MSE	EV	MSE
2	30	15	(15, 0*14)	1.5993	0.1166	0.9163	0.0986	0.5701	0.1019
			(0*6, 6, 5, 4, 0*6)	1.5977	0.1187	0.9200	0.0936	0.5684	0.0982
			(0*14, 15)	1.5968	0.1098	1.0769	0.0942	0.5675	0.0951
		20	(10, 0*19)	1.5887	0.0990	0.9287	0.0860	0.5793	0.0878
			(1, 0)*10	1.5847	0.0948	0.9334	0.0841	0.5682	0.0865
			(0*19, 10)	1.5822	0.0908	0.9523	0.0759	0.5579	0.0769
		30	(0*30)	1.5412	0.0703	0.9589	0.0712	0.5463	0.0753
	50	25	(25, 0*24)	1.5582	0.0928	0.9309	0.0854	0.5586	0.0824
			(0*8, 1, 3*8, 0*8)	1.5647	0.0910	1.0642	0.0819	0.5579	0.0821
			(0*24, 25)	1.5630	0.0904	0.9397	0.0810	0.5565	0.0802
		30	(20, 0*29)	1.5582	0.0793	0.9518	0.0746	0.5490	0.0774
			(2, 0, 0)*10	1.5451	0.0758	0.9546	0.0728	0.5446	0.0727
			(0*29, 20)	1.5432	0.0726	0.9567	0.0724	0.5472	0.0715
		50	(0*50)	1.5307	0.0701	0.9643	0.0704	0.5437	0.0738
3	30	15	(15, 0*14)	1.5871	0.1132	0.9274	0.0932	0.5893	0.0906
			(0*6, 6, 5, 4, 0*6)	1.5834	0.1124	0.9286	0.0915	0.5648	0.0874
			(0*14,15)	1.5823	0.1052	0.9351	0.0913	0.5658	0.0857
		20	(10, 0*19)	1.5775	0.0987	0.9487	0.0783	0.5522	0.0795
			(1, 0)*10	1.5744	0.0943	0.9451	0.0780	0.5489	0.0778
			(0*19,10)	1.5716	0.0891	0.9488	0.0743	0.5533	0.0766
		30	(0*30)	1.5401	0.0701	0.9591	0.0709	0.5430	0.0742
	50	25	(25, 0*24)	1.5435	0.0922	0.9491	0.0817	0.5527	0.0763
			(0*8, 1, 3*8, 0*8)	1.5682	0.0906	0.9524	0.0745	0.5518	0.0751
			(0*24,25)	1.5479	0.0897	0.9569	0.0737	0.5576	0.0744
		30	(20, 0*29)	1.5474	0.0762	1.0380	0.0678	0.5443	0.0742
			(2,0,0)*10	1.5446	0.0754	0.9680	0.0665	0.5563	0.0620
			(0*29,20)	1.5419	0.0719	0.9682	0.0689	0.5541	0.0687
		50	(0*50)	1.5209	0.0643	0.9688	0.0702	0.5413	0.0730

**Table 3:** Bayesian estimates and MSEs of the parameters under GELF when q=−0.5,α=1.5,β=1,λ=0.5,M=2000.

*k*	*n*	*m*	Censoring Scheme	${\hat{\alpha }}_{\mathrm{BG}}$		${\hat{\beta }}_{\mathrm{BG}}$		${\hat{\lambda }}_{\mathrm{BG}}$	
				EV	MSE	EV	MSE	EV	MSE
2	30	15	(15, 0*14)	1.5985	0.1165	0.9170	0.0986	0.5673	0.1004
			(0*6, 6, 5, 4, 0*6)	1.5965	0.1183	0.9213	0.0935	0.5640	0.0976
			(0*14,15)	1.5951	0.1095	1.0761	0.0940	0.5621	0.0950
		20	(10, 0*19)	1.5872	0.0988	0.9294	0.0858	0.5779	0.0871
			(1, 0)*10	1.5841	0.0941	0.9346	0.0840	0.5677	0.0859
			(0*19,10)	1.5818	0.0902	0.9529	0.0761	0.5548	0.0757
		30	(0*30)	1.5407	0.0701	0.9593	0.0710	0.5458	0.0751
	50	25	(25, 0*24)	1.5573	0.0923	0.9327	0.0852	0.5580	0.0822
			(0*8, 1, 3*8, 0*8)	1.5640	0.0907	1.0651	0.0815	0.5558	0.0814
			(0*24, 25)	1.5625	0.0901	0.9427	0.0805	0.5549	0.0801
		30	(20, 0*29)	1.5572	0.0790	0.9536	0.0741	0.5487	0.0772
			(2, 0, 0)*10	1.5441	0.0752	0.9551	0.0723	0.5438	0.0724
			(0*29, 20)	1.5428	0.0723	0.9574	0.0720	0.5469	0.0711
		50	(0*50)	1.5278	0.0696	0.9650	0.0702	0.5430	0.0736
3	30	15	(15, 0*14)	1.5863	0.1128	0.9289	0.0930	0.5887	0.0904
			(0*6, 6, 5, 4, 0*6)	1.5825	0.1120	0.9294	0.0914	0.5632	0.0869
			(0*14, 15)	1.5812	0.1048	0.9378	0.0911	0.5652	0.0857
		20	(10, 0*19)	1.5763	0.0981	0.9490	0.0782	0.5516	0.0794
			(1, 0)*10	1.5739	0.0939	0.9459	0.0778	0.5478	0.0773
			(0*19, 10)	1.5710	0.0882	0.9492	0.0740	0.5527	0.0764
		30	(0*30)	1.5352	0.0693	0.9598	0.0707	0.5428	0.0740
	50	25	(25, 0*24)	1.5426	0.0917	0.9521	0.0813	0.5515	0.0761
			(0*8, 1, 3*8, 0*8)	1.5620	0.0901	0.9534	0.0741	0.5505	0.0748
			(0*24,25)	1.5468	0.0892	0.9578	0.0735	0.5570	0.0744
		30	(20, 0*29)	1.5461	0.0759	1.0387	0.0677	0.5437	0.0740
			(2, 0, 0)*10	1.5438	0.0751	0.9689	0.0663	0.5556	0.0619
			(0*29, 20)	1.5401	0.0712	0.9680	0.0685	0.5532	0.0683
		50	(0*50)	1.5209	0.0640	0.9694	0.0701	0.5410	0.0729

**Table 4:** Bayesian estimates and MSEs of the parameters under GELF when q=0.5,α=1.5,β=1,λ=0.5,M=2000.

*k*	*n*	*m*	Censoring Scheme	${\hat{\alpha }}_{\mathrm{BG}}$		${\hat{\beta }}_{\mathrm{BG}}$		${\hat{\lambda }}_{\mathrm{BG}}$	
				EV	MSE	EV	MSE	EV	MSE
2	30	15	(15, 0*14)	1.5974	0.1163	0.9271	0.0964	0.5658	0.1003
			(0*6, 6, 5, 4, 0*6)	1.5948	0.1181	0.9264	0.0935	0.5634	0.0974
			(0*14, 15)	1.5950	0.1095	1.0741	0.0938	0.5617	0.0931
		20	(10, 0*19)	1.4254	0.0982	0.9303	0.0853	0.5717	0.0869
			(1, 0)*10	1.4269	0.0940	0.9366	0.0840	0.5665	0.0854
			(0*19, 10)	1.4287	0.0901	0.9529	0.0760	0.5538	0.0751
		30	(0*30)	1.5421	0.0703	0.9602	0.0705	0.5421	0.0750
	50	25	(25, 0*24)	1.5564	0.0921	0.9341	0.0852	0.5568	0.0820
			(0*8, 1, 3*8, 0*8)	1.5636	0.0905	1.0649	0.0812	0.5561	0.0814
			(0*24, 25)	1.5609	0.0899	0.9446	0.0804	0.5540	0.0796
		30	(20, 0*29)	1.5572	0.0790	0.9537	0.0740	0.5482	0.0771
			(2, 0, 0)*10	1.5438	0.0750	0.9542	0.0721	0.5434	0.0724
			(0*29, 20)	1.5419	0.0720	0.9556	0.0719	0.5468	0.0710
		50	(0*50)	1.5267	0.0695	0.9657	0.0701	0.5432	0.0736
3	30	15	(15, 0*14)	1.5856	0.1124	0.9313	0.0927	0.5849	0.0902
			(0*6, 6, 5, 4, 0*6)	1.5816	0.1118	0.9345	0.0912	0.5621	0.0863
			(0*14,15)	1.5803	0.1042	0.9397	0.0910	0.5638	0.0853
		20	(10, 0*19)	1.5758	0.0980	0.9490	0.0782	0.5516	0.0794
			(1, 0)*10	1.5727	0.0935	0.9459	0.0778	0.5478	0.0773
			(0*19,10)	1.5704	0.0880	0.9492	0.0740	0.5527	0.0764
		30	(0*30)	1.5348	0.0691	0.9598	0.0707	0.5428	0.0740
	50	25	(25, 0*24)	1.5417	0.0912	0.9521	0.0813	0.5515	0.0761
			(0*8, 1, 3*8, 0*8)	1.5607	0.0897	0.9534	0.0741	0.5505	0.0748
			(0*24, 25)	1.5457	0.0889	0.9578	0.0735	0.5570	0.0744
		30	(20, 0*29)	1.5457	0.0754	1.0387	0.0677	0.5437	0.0740
			(2, 0, 0)*10	1.5427	0.0749	0.9689	0.0663	0.5456	0.0615
			(0*29, 20)	1.5376	0.0707	0.9680	0.0685	0.5432	0.06831
		50	(0*50)	1.5203	0.0636	0.9663	0.0697	0.5249	0.0721

**Table 5:** Bayesian estimates and MSEs of the parameters under GELF when q=1,α=1.5,β=1,λ=0.5,M=2000.

*k*	*n*	*m*	Censoring Scheme	${\hat{\alpha }}_{\mathrm{BG}}$		${\hat{\beta }}_{\mathrm{BG}}$		${\hat{\lambda }}_{\mathrm{BG}}$	
				EV	MSE	EV	MSE	EV	MSE
2	30	15	(15, 0*14)	1.5971	0.1162	0.9268	0.0962	0.5651	0.1002
			(0*6, 6, 5, 4, 0*6)	1.5929	0.1178	0.9281	0.0934	0.5604	0.0972
			(0*14, 15)	1.5938	0.1090	1.0732	0.0929	0.5597	0.0934
		20	(10, 0*19)	1.4342	0.0980	0.9379	0.0850	0.5703	0.0867
			(1, 0)*10	1.4381	0.0932	0.9398	0.0831	0.5657	0.0850
			(0*19, 10)	1.4379	0.0895	0.9563	0.0759	0.5516	0.0750
		30	(0*30)	1.5412	0.0702	0.9638	0.0702	0.5419	0.0748
	50	25	(25, 0*24)	1.5547	0.0918	0.9386	0.0850	0.5545	0.0818
			(0*8, 1, 3*8, 0*8)	1.5624	0.0904	1.0627	0.0810	0.5549	0.0811
			(0*24, 25)	1.5601	0.0896	0.9458	0.0802	0.5527	0.0792
		30	(20, 0*29)	1.5553	0.0787	0.9549	0.0738	0.5458	0.0768
			(2, 0, 0)*10	1.5431	0.0749	0.9538	0.0720	0.5428	0.0722
			(0*29, 20)	1.5416	0.0719	0.9552	0.0720	0.5467	0.0706
		50	(0*50)	1.5264	0.0695	0.9671	0.0700	0.5428	0.0734
3	30	15	(15, 0*14)	1.5827	0.1117	0.9348	0.0924	0.5827	0.0901
			(0*6, 6, 5, 4, 0*6)	1.5801	0.1116	0.9431	0.0910	0.5618	0.0861
			(0*14, 15)	1.5801	0.1042	0.9416	0.0907	0.5626	0.0850
		20	(10, 0*19)	1.5736	0.0978	0.9512	0.0778	0.5510	0.0792
			(1, 0)*10	1.5727	0.0935	0.9459	0.0778	0.5478	0.0773
			(0*19,10)	1.5701	0.0880	0.9531	0.0739	0.5520	0.0764
		30	(0*30)	1.5327	0.0690	0.9632	0.0705	0.5423	0.0739
	50	25	(25, 0*24)	1.5410	0.0910	0.9536	0.0811	0.5501	0.0760
			(0*8, 1, 3*8, 0*8)	1.5601	0.0897	0.9528	0.0740	0.5497	0.0743
			(0*24, 25)	1.5426	0.0883	0.9590	0.0733	0.5537	0.0742
		30	(20, 0*29)	1.5419	0.0752	1.0378	0.0674	0.5432	0.0740
			(2, 0, 0)*10	1.5412	0.0746	0.9697	0.0661	0.5447	0.0612
			(0*29, 20)	1.5354	0.0705	0.9694	0.0683	0.5420	0.0631
		50	(0*50)	1.5202	0.0635	0.9768	0.0694	0.5238	0.0718

**Table 6:** The average length (AL) and coverage probability (CP) of 95% asymptotic confidence interval for parameters when α=1.5,β=1,λ=0.5,M=2000.

*k*	*n*	*m*	Censoring Scheme	${\hat{\alpha }}_{\mathrm{ACI}}$		${\hat{\beta }}_{\mathrm{ACI}}$		${\hat{\lambda }}_{\mathrm{ACI}}$	
				AL	CP	AL	CP	AL	CP
2	30	15	(15, 0*14)	2.1359	0.945	1.6560	0.944	1.2398	0.948
			(0*6, 6, 5, 4, 0*6)	2.0936	0.943	1.6849	0.942	1.2356	0.947
			(0*14, 15)	2.0528	0.943	1.7936	0.951	1.2125	0.945
		20	(10, 0*19)	1.9267	0.946	1.5312	0.949	1.1243	0.950
			(1, 0)*10	1.9587	0.948	1.7287	0.952	1.1183	0.951
			(0*19, 10)	1.8942	0.943	1.5242	0.946	1.1146	0.949
		30	(0*30)	1.9051	0.953	1.5351	0.952	1.1048	0.955
	50	25	(25, 0*24)	1.8797	0.955	1.5617	0.948	1.1118	0.953
			(0*8, 1, 3*8, 0*8)	1.8415	0.952	1.5425	0.945	1.0581	0.952
			(0*24, 25)	1.8344	0.951	1.2889	0.946	1.0024	0.951
		30	(20, 0*29)	1.6577	0.958	1.1018	0.951	0.9189	0.952
			(2, 0, 0)*10	1.6134	0.956	1.5134	0.957	0.9664	0.959
			(0*29, 20)	1.5581	0.953	1.1980	0.954	0.8893	0.955
		50	(0*50)	1.5128	0.957	1.4651	0.959	0.9246	0.957
3	30	15	(15, 0*14)	1.7536	0.948	1.1076	0.947	1.0056	0.948
			(0*6, 6, 5, 4, 0*6)	1.7625	0.945	1.0431	0.945	0.9834	0.947
			(0*14, 15)	1.7560	0.942	1.6560	0.954	0.9062	0.945
		20	(10, 0*19)	1.5921	0.951	0.9611	0.949	0.8753	0.950
			(1, 0)*10	1.6313	0.953	0.9661	0.954	0.8766	0.951
			(0*19, 10)	1.5442	0.952	1.3442	0.956	0.8043	0.949
		30	(0*30)	1.5956	0.955	1.5247	0.959	0.8016	0.956
	50	25	(25, 0*24)	1.5068	0.956	0.9455	0.951	0.7643	0.953
			(0*8, 1, 3*8, 0*8)	1.5082	0.954	0.8636	0.948	0.7743	0.952
			(0*24, 25)	1.4889	0.952	1.2728	0.947	0.7169	0.951
		30	(20, 0*29)	1.4786	0.960	0.9245	0.951	0.6731	0.952
			(2, 0, 0)*10	1.4391	0.957	0.8545	0.957	0.6817	0.957
			(0*29, 20)	1.3980	0.954	1.1273	0.959	0.6290	0.955
		50	(0*50)	1.3879	0.961	1.1348	0.958	0.6203	0.959

**Table 7:** The average length (AL) and coverage probability (CP) of 95% HPD credible intervals for parameters when α=1.5,β=1,λ=0.5,M=2000.

*k*	*n*	*m*	Censoring Scheme	${\hat{\alpha }}_{\mathrm{HPD}}$		${\hat{\beta }}_{\mathrm{HPD}}$		${\hat{\lambda }}_{\mathrm{HPD}}$	
				AL	CP	AL	CP	AL	CP
2	30	15	(15, 0*14)	1.9507	0.946	1.3070	0.951	1.1772	0.951
			(0*6, 6, 5, 4, 0*6)	1.9249	0.945	1.3122	0.951	1.1803	0.952
			(0*14, 15)	1.8799	0.944	1.2852	0.950	1.1423	0.948
		20	(10, 0*19)	1.7347	0.9 51	1.1515	0.952	1.0889	0.952
			(1, 0)*10	1.7034	0.948	1.2790	0.955	1.0766	0.953
			(0*19, 10)	1.6723	0.949	1.1328	0.951	1.0496	0.956
		30	(0*30)	1.6549	0.958	1.1258	0.954	1.0467	0.956
	50	25	(25, 0*24)	1.5696	0.956	1.0608	0.951	0.9954	0.953
			(0*8, 1, 3*8, 0*8)	1.5726	0.954	1.1023	0.949	0.9831	0.954
			(0*24, 25)	1.4319	0.958	1.0281	0.947	0.9068	0.952
		30	(20, 0*29)	1.3533	0.961	0.9863	0.952	0.8466	0.954
			(2, 0, 0)*10	1.4284	0.962	1.0047	0.959	0.8629	0.960
			(0*29, 20)	1.2657	0.956	0.9678	0.955	0.8223	0.956
		50	(0*50)	1.2657	0.959	0.9789	0.960	0.8341	0.960
3	30	15	(15, 0*14)	1.4718	0.951	0.9802	0.948	0.8865	0.950
			(0*6, 6, 5, 4, 0*6)	1.4972	0.953	0.9927	0.950	0.9472	0.951
			(0*14, 15)	1.3936	0.949	0.9172	0.954	0.8474	0.949
		20	(10, 0*19)	1.3215	0.953	0.9064	0.951	0.7753	0.952
			(1, 0)*10	1.3459	0.956	0.8943	0.956	0.8202	0.953
			(0*19, 10)	1.2881	0.952	0.8298	0.957	0.7546	0.956
		30	(0*30)	1.3552	0.957	0.8762	0.961	0.7813	0.961
	50	25	(25, 0*24)	1.1733	0.959	0.8194	0.954	0.7656	0.958
			(0*8, 1, 3*8, 0*8)	1.2339	0.957	0.8166	0.950	0.7388	0.953
			(0*24, 25)	1.1756	0.961	0.7711	0.953	0.6823	0.952
		30	(20, 0*29)	1.0191	0.961	0.6264	0.953	0.6643	0.954
			(2, 0, 0)*10	1.0989	0.958	0.6845	0.959	0.6743	0.959
			(0*29, 20)	0.9535	0.956	0.6620	0.960	0.6619	0.961
		50	(0*50)	0.9672	0.963	0.6798	0.963	0.6597	0.959

**Table 8:** The fitting results for the real data set of survival times of 72 guinea pigs data.

Distribution	MLEs	AIC	CAIC	BIC	HQIC	K-S
IPLD	$\begin{array}{l} \hat{\alpha }=0.6971,\hat{\lambda }=0.1302\\ \hat{\beta }=3.3638 \end{array}$	193.0546	193.3983	199.8854	195.7738	0.0743
LD	$\hat{\alpha }=182.4212,\hat{\lambda }=103.5113$	230.5347	230.7038	235.0892	232.3482	0.6904
ELD	$\begin{array}{l} \hat{\alpha }=3.7163,\lambda =0.0151,\\ \hat{\theta }=78.3218 \end{array}$	194.5692	194.9124	201.3987	197.2882	0.0941
PLD	$\begin{array}{l} \hat{\alpha }=1.7087,\hat{\lambda }=6.1974,\\ \hat{\beta }=2.5742 \end{array}$	193.0753	193.4182	199.9052	195.7943	0.0782
IWD	$\hat{\alpha }=1.0692,\hat{\lambda }=1.1734$	240.3324	240.5014	244.8854	242.1453	0.1968
GIWD	$\begin{array}{l} \hat{\alpha }=0.1089,\hat{\gamma }=14.3738,\\ \hat{\beta }=1.1732 \end{array}$	242.3318	242.6753	249.1618	245.0512	0.1973
ILD	$\hat{\alpha }=12.9073,\hat{\beta }=0.0958$	242.8217	242.9958	247.3747	244.6346	0.9986

**Table 9:** Progressive first-failure censored samples under the given censoring schemes when *k* = 2, *n* = 36, *m* = 26.

Censoring Scheme	Progressive First-Failure Censored Sample
CS1 = (10, 0*25)	0.1, 1.2, 1.22, 1.24, 1.4, 1.34, 1.39, 1.46, 1.59, 1.63, 1.68, 1.72, 1.83, 1.97, 2.02, 2.15, 2.22, 2.31, 2.45, 2.53, 2.54, 2.78, 2.93, 3.42, 3.61, 4.02.
CS2 = (0*11, 3,4,3, 0*12)	0.1, 0.44, 0.59, 0.74, 0.93, 1, 1.05, 1.07, 1.08, 1.12, 1.15, 1.2, 1.22, 1.39, 1.72, 2.15, 2.22, 2.31, 2.45, 2.53, 2.54, 2.78, 2.93, 3.42, 3.61, 4.02.
CS3 = (0*25, 10)	0.1, 0.44, 0.59, 0.74, 0.93, 1, 1.05, 1.07, 1.08, 1.12, 1.15, 1.2, 1.22, 1.24, 1.4, 1.34, 1.39, 1.46, 1.59, 1.63, 1.68, 1.72, 1.83, 1.97, 2.02, 2.15

**Table 10:** MLEs and Bayesian estimations (BEs) of parameters for the real data sets under different censoring scheme.

MLEs	Censoring Schemes			BEs (Squared Loss)	Censoring Schemes		
	CS1	CS2	CS3		CS1	CS2	CS3
${\hat{\alpha }}_{ML}$	0.8245	0.4263	0.5248	${\hat{\alpha }}_{\mathrm{BS}}$	0.8168	0.4375	0.5357
${\hat{\beta }}_{ML}$	0.1982	0.0721	0.1156	${\hat{\beta }}_{\mathrm{BS}}$	0.1893	0.0786	0.1274
${\hat{\lambda }}_{ML}$	4.1089	2.3716	2.4823	${\hat{\lambda }}_{\mathrm{BS}}$	4.1025	2.3785	2.4969

**Table 11:** Bayesian estimations of parameters under GELF.

BEs Entropy Loss	q=−0.5			q=0.5			q=1		
	Censoring Schemes			Censoring Schemes			Censoring Schemes		
	CS1	CS2	CS3	CS1	CS2	CS3	CS1	CS2	CS3
${\hat{\alpha }}_{\mathrm{BG}}$	0.8472	0.4236	0.5318	0.8147	0.4380	0.5366	0.8025	0.4453	0.5354
${\hat{\beta }}_{\mathrm{BG}}$	0.1927	0.0735	0.1298	0.1894	0.0792	0.1289	0.1823	0.0786	0.1274
${\hat{\lambda }}_{\mathrm{BG}}$	4.1354	2.3692	2.4987	4.1016	2.3775	2.4977	4.1025	2.3785	2.4969

**Table 12:** The 95% asymptotic confidence intervals (ACIs) and HPD credible intervals HPDCIs of the parameters.

Parameter	ACIs			Parameter	HPDCIs		
	CS1	CS2	CS3		CS1	CS2	CS3
α	(0.2426, 2.4109)	(0.1917, 1.5328)	(0.1879, 2.1357)	α	(0.2426, 2.4103)	(0.1931, 1.5319)	(0.1884, 2.1352)
β	(0.0943, 1.8561)	(0.0257, 1.3771)	(0.0876,1.7457)	β	(0.0950, 1.8546)	(0.0265, 1.3762)	(0.0882,1.7451)
λ	(0.8465,5.8102)	(0.5413, 3.1485)	(0.6874, 3.5438)	λ	(0.8479, 5.8068)	(0.5620, 3.1424)	(0.6892, 3.5416)

## Data Availability

The data are available from the corresponding author upon request.
